# Water Bath Scallop Shucking System Based on Doneness Detection

**DOI:** 10.3390/s26144545

**Published:** 2026-07-17

**Authors:** Guoliang Yang, Xiangnian Shang, Kai Cheng

**Affiliations:** 1 Faculty of Information Science and Engineering, Ocean University of China, Qingdao 266100, China; y2961318319@163.com (G.Y.); chengkai@ouc.edu.cn (K.C.); 2 Engineering Research Center of Advanced Marine Physical Instruments and Equipment, Ministry of Education, Qingdao 266100, China

**Keywords:** scallop shucking, visual feedback, doneness detection, RT-DETR, temperature control, deep learning

## Abstract

In the scallop shucking industry, labor-intensive manual operations and inconsistent product quality remain persistent challenges. To address these issues, this study develops a scallop shucking system based on visual feedback temperature control. To support this system, systematic experiments are conducted to determine the baseline shucking method and its initial parameters. On this basis, the scallop doneness detection model SDD-RT-DETR, serving as the system’s core, integrates an enhanced backbone centered on the self-developed module HierarchicalRepBlock, a frequency-domain self-attention module AIFI-EDFFN, a neck featuring the self-developed EfficientBalanceFusion module as the feature fusion unit and the Converse2DC3 module as the feature extraction unit, and the Wise-DIoU loss function. A scallop doneness image dataset specifically constructed for this task was used to train and validate this model. Experimental results demonstrate that the model achieves 95.5% accuracy, 93.6% recall, and 96.1% mAP50, improving by 4.7%, 3.2%, and 4.2%, respectively, over the baseline model RT-DETR. Additionally, the model’s computational cost was reduced by 18.9%, and its parameters were reduced by 14.1% compared to the baseline model. This model provides real-time, accurate doneness assessment, thereby filling a gap in computer vision for scallop doneness detection. Furthermore, this study integrated this model into a water bath scallop shucking system with feedback temperature control, ensuring that the doneness of scallops after shucking is maintained within an optimal range. Batch tests show that this system achieves a 96.6% shucking rate and an 88.5% properly cooked rate, outperforming the conventional method. This improves the quality of automatically shucked scallops while offering a practical solution for the intelligent upgrading of the seafood processing industry.

## 1. Introduction

As an important commercial shellfish, scallops are highly favored by consumers for their sweet, tender meat and rich nutritional value [1]. Growing market demand has driven rapid expansion in the output value and scale of the scallop aquaculture industry [2]. However, behind the industry’s booming development, the supporting scallop processing segment has failed to keep pace. In particular, the shucking process still relies heavily on manual labor, resulting in low efficiency, high costs, and inconsistent product quality, which hinders the industry’s automation and scalability [3,4]. Consequently, developing a manual-free shucking method that ensures consistent product quality has become a critical challenge for the industry.

To address this issue, researchers have explored various shucking methods, with thermal shucking and ultra-high-pressure shucking being the most representative examples. In 1961, Bullis et al. invented a steam shucking machine for scallops that achieved good results [5], which involves first subjecting the scallops to a steam jet, with subsequent processing facilitated by vacuum application for cleaning. Martin et al. developed a thermal/cold treatment process for efficient shucking [6], which involves heating via steam injection followed by rapid cooling with an ice-water solution to achieve efficient separation of the shell and meat. He and Adam et al. conducted high-pressure shucking tests on shellfish within a pressure range of 207–310 MPa [7], and the results showed that high-pressure treatment can effectively open scallop shells. Kim et al. investigated a novel ultra-high-pressure shucking method for shellfish [8], which involves applying 150–250 MPa of hydrostatic pressure to a sealed pressure vessel to induce rapid, automatic separation of the shell and meat. The scallop meat processed using this method is tender and of high quality.

However, although ultra-high-pressure shucking achieves high-quality results, it is severely limited by high equipment costs and discontinuous processing, posing significant challenges for industrial application [9]. The current mainstream automated shucking method still predominantly relies on a steam jet. While this method offers good shucking performance and efficiency, the high-temperature steam typically used in actual production often leads to overcooking of the scallops. Overcooked scallops exhibit shriveled meat and poor texture, with significant loss of umami and nutritional components [10], rendering them unsuitable as semi-finished material for subsequent processing and severely affecting their commercial value.

To address these issues, this study, through experimental comparisons of existing thermal shucking methods (see Section 2.1.1 for details), identified low-temperature (75–90 °C) water bath shucking as the baseline method. However, the experiments (see Section 2.1.2 for details) show that even when using this method, the shucking parameters could not be kept constant without sacrificing quality. Factors such as the holding time after harvest, the time to market, and size variations among different batches all influence the optimal shucking parameters, with holding time being the most significant (the longer scallops are held, the lower their vitality [11]; the physiological states of scallops vary significantly across seasons [12]; and larger scallops have more developed adductor muscles [13]). Therefore, if the water bath parameters for shucking are fixed, it cannot adapt to changes in the condition of the scallops, potentially leading to issues such as incomplete shucking or overcooked meat. Although it is possible to manually adjust the water bath parameters based on the condition of the scallops, this approach has two main drawbacks: first, it consumes significant manpower and resources, preventing full automation of the production line; second, the effectiveness of adjustments relies heavily on the operator’s experience and subjective judgment, making it difficult to ensure consistent quality. Based on these requirements, this study aims to develop a scallop doneness detection model. By embedding the model into the low-temperature water bath shucking system as a feedback unit, it enables automatic adjustment of the water bath parameters, thus achieving vision feedback temperature control.

The detection model is responsible for identifying scallops on the production line and providing information on their degree of doneness. It serves as the core of this system and must meet extremely high standards for both detection accuracy and speed. In recent years, rapid advancements in neural networks and computer vision have led to the emergence of numerous object detection models. Examples include two-stage detection models such as R-CNN [14], Fast R-CNN [15], and Mask R-CNN [16]; single-stage detection models such as the SSD series [17] and YOLO series [18]; as well as Transformer-based models such as Vision Transformer [19], Swin Transformer [20], DETR [21], RT-DETR [22], and DINO [23]. And these models have been increasingly adopted in intelligent agricultural and aquacultural automation. Kuswantori et al. developed a fish classification system for automatic sorting based on YOLOv4 [24], achieving 98.15% accuracy on a dataset of eight freshwater fish species. Mainali and Li developed a robotic fish processing line enhanced by machine learning [25], including fish identification by type, sorting by size, orientation based on shape, and cutting at optimal chopping points. Huang and Wei proposed an algorithm for sea cucumber recognition and sorting based on improved YOLOv9 and RepViT [26], which achieves an accuracy of 98.33% on their self-built dataset. Zhang et al. proposed a lightweight detection model based on YOLOv11 [27], which was designed for automated shrimp meat quality control, achieving 82.1% mAP50 on the PASCAL VOC dataset. Hurst et al. designed a robotic system for sorting crustaceans [28], consisting of a waterproof robotic arm with a custom caging gripper and a YOLOv5-OBB-based vision system for crayfish detection and evaluation has shown that the system can recognize and select crayfish without causing any damage. Han et al. proposed SEAF-Net, a lightweight detection model based on YOLOv11 designed for low-contrast and highly dynamic underwater environments [29], which achieves an mAP50 of 93.333% on a standardized 13-class underwater fish dataset. Shimamoto et al. developed an autonomous underwater drone system that integrates a fish school detection model based on YOLOv8 with navigation control [30], enabling the drone to track fish based on the detection results. Beyond these applications, several studies have also focused on the specific field of scallop detection. For two-stage detection models, Zhang et al. proposed a shellfish recognition model named FL_Net based on CNN [31], which achieved an accuracy of 93.95% on their self-built dataset. To address the dynamic detection needs of multiple and overlapping targets in practical scenarios, Feng et al. proposed a shellfish detection algorithm based on an improved Faster R-CNN [32], which successfully reduces missed detections of dense or occluded targets. Regarding the application of single-stage detection algorithms in scallop detection, Li et al. proposed an underwater scallop recognition algorithm based on improved YOLOv5s [33], providing an effective solution for the automated identification of bottom-sown scallops. Furthermore, to address challenges such as poor image quality and missed detection of small targets during underwater harvesting, Qian et al. proposed the WDS-YOLO detection model based on improved YOLOv8n [34], offering an important reference for the accurate detection of multiple types of marine benthos, including scallops. Based on the Transformer structure, Rao et al. proposed the benthic organism detection network Benthos-DETR [35], offering an end-to-end solution for the high-precision identification of various underwater organisms, including scallops. In summary, current deep learning research on scallop detection has established a relatively comprehensive technical framework, with significant progress achieved in high-precision identification, size classification, and multi-object detection in complex underwater environments. However, existing work primarily focuses on the identification and localization of unopened scallops, leaving scallop doneness, a critical indicator of quality and processing status, as an unexplored area.

For shucked scallops, accurately determining their doneness requires the use of fine-grained features such as color difference and texture. Directly applying general detection models makes it difficult to capture such subtle differences; therefore, the model must be designed with a targeted structure to enhance its capability to capture fine-grained features. Furthermore, shucked scallops on the production line tend to pile up, requiring the model to be optimized for dense distributions and occlusions. Additionally, when captured from a higher position, scallop targets appear relatively small, meaning the model’s capability to detect small objects must be taken into account. Finally, considering that the model will be deployed on embedded devices and high-speed production lines, the model must be lightweight, and it must meet high inference speed requirements. To address these challenges, this study presents SDD-RT-DETR, a scallop doneness detection model based on improved RT-DETR for integration into a shucking system to provide doneness information. Moreover, this model can be extended to subsequent scallop sorting and grading tasks, providing technical support for the automated assessment of scallop quality.

By integrating SDD-RT-DETR into the low-temperature water bath scallop shucking system with feedback temperature control, the system aims to continuously maintain the product’s properly cooked rate within the optimal range without manual intervention, thereby driving the transformation of traditional scallop shucking toward intelligent and standardized shucking.

In summary, the main contributions of this work are as follows:

(1) Through systematic experiments on water bath scallop shucking, we investigated the effects of key parameters (e.g., temperature, soaking time) on shucking success rate, doneness, and adductor muscle tension.
(2) A scallop image dataset covering various doneness states was constructed to support the subsequent model’s precise learning of different states.
(3) A real-time object detection model named SDD-RT-DETR is designed as the core feedback component of the system, capable of accurately locating scallops and determining their doneness level.
(4) A low-temperature water bath scallop shucking system was developed, which integrates the scallop doneness detection model SDD-RT-DETR with a precision feedback temperature control unit.

## 2. Materials and Methods

### 2.1. Scallop Shucking Experiments

To determine an appropriate shucking method and corresponding parameters, as well as to define doneness classification criteria, this study conducted systematic experiments on scallop shucking. This work primarily consists of the following sections: comparative experiments of thermal treatment methods; experiments on the effects of water bath parameters on scallop shucking performance and doneness; and experiments on the effects of water bath treatment on adductor muscle tension. The experimental material was bay scallops sourced from coastal aquaculture areas in Qingdao, Shandong Province, China. As a locally dominant economic species, the bay scallop is farmed on a large scale with stable yield, making it the single highest yielding species among farmed scallops worldwide [36]. Using this species as the research subject adequately reflects the commonality of raw materials in real processing production, ensuring that the experimental conclusions provide practical guidance for the scallop processing industry. And all experiments were conducted during the peak scallop harvest season (mid-to-late November).

#### 2.1.1. Comparative Experiments of Thermal Treatment Methods

This section compares several common thermal treatment methods to identify the most suitable baseline approach. First is the direct heating method, which uses an alcohol burner to heat the scallops. The shucking rate is generally low, making it difficult to meet stable industrial shucking standards; simultaneously, the flame temperature is difficult to control, which can easily cause localized burns to the adductor muscle, affecting quality.

The water jet shucking is another widely used method. Experimental results show that scallops can be shucked in 9–13 s at 90 °C; when the water temperature rises to 100 °C, the shucking time can be reduced to 4–7 s. Although this method has a high shucking efficiency, tests indicate that a 5 s treatment can raise the internal shell temperature to 60–80 °C, often leading to overcooked scallop meat. Furthermore, this method consumes a vast amount of water, posing significant water resource consumption issues in large-scale production.

The steam jet shucking also demonstrated good results in experiments and is highly efficient, typically completing the process within 5–10 s. However, this method is similarly prone to overcooking the scallop meat and requires strict equipment control, requiring precise adjustment of multiple parameters such as jetting time, distance, and steam displacement. Its high operational complexity does not meet the real-time control requirements for the subsequent stages of this study.

Low-temperature water bath shucking was selected as the final baseline method. Compared to other thermal treatment methods, this method can complete shucking in a brief time at a lower temperature (see Section 2.1.2 for details). While ensuring high shucking efficiency, this method effectively alleviates meat overcooking caused by high temperatures and avoids the complexity of coordinating multiple parameters. Thus, this method meets the comprehensive demands of industrial production regarding efficiency, cost, and quality control, thereby fulfilling the requirements of this study.

#### 2.1.2. Experiments on the Effects of Water Bath Parameters

This section primarily investigates the effects of water bath parameters on scallop shucking performance and doneness. Before the experiments, scallops were pretreated in a 30 °C water bath for approximately 20 min to relax the adductor muscle. Subsequently, batch tests were conducted in a constant-temperature bath with various combinations of temperature and time. The results indicate that for scallops held for no more than 2 h after harvesting, the optimal temperature range is 83–87 °C. Within this temperature range, shucking time is short (5–20 s) and can effectively prevent overcooking of the scallop meat. When the temperature is below 80 °C, the shucking time is significantly prolonged, and the adductor muscle tends to adhere to the shell, making it difficult for the adductor muscle to detach naturally; whereas when the temperature exceeds 90 °C, although shucking can be completed in a short time, the adductor muscle shows obvious signs of overcooking, and the mantle shrinks significantly, affecting overall quality. The shucking time and sensory evaluation at different water bath temperatures are shown in Table 1.

It should be noted that the above results were obtained under controlled conditions using scallops of consistent freshness. In actual production, scallop conditions vary considerably in terms of freshness, size variation across batches, and species, all of which may affect shucking performance. Based on our considerable experimental experience over the past several years, we have found that seasonal and species-related differences mainly affect the initial shucking parameters and do not require real-time adjustment once the baseline is determined (the subsequent feedback system can determine them with a single adjustment), whereas the post-harvest holding time (freshness) is considered the most critical factor, as it varies continuously and directly determines the relaxation state of the adductor muscle, thus requiring real-time adjustment of the shucking parameters. Therefore, we further conducted experiments on the effects of post-harvest holding time on shucking performance and scallop doneness. The scallops for this experiment were held at room temperature for approximately 2, 3, and 4 h, respectively. After pretreatment in a 30 °C water bath for about 20 min, experiments were conducted within the previously determined optimal temperature range (83–87 °C) and adjacent temperatures.

Under the same 85 °C water bath conditions, scallops held for approximately 2 h could be shucked within 5–20 s, with good meat quality. Whereas scallops held for approximately 3 h required 15–40 s to be shucked. The extended processing time caused some samples to exhibit mantle contraction and obvious surface denaturation of the adductor muscle. When the holding time was extended to approximately 4 h, even with a processing time of over 60 s, natural detachment of the adductor muscle remained difficult for some samples, and prolonged thermal treatment had already caused a distinct cooked layer on the adductor muscle.

To address these issues, we attempted to compensate by increasing the processing temperature. The results showed that for scallops held for approximately 3 h, raising the water bath temperature to 88 °C allowed for shucking within 5–20 s, while the reduction in processing time effectively prevents overcooking of the adductor muscle. For scallops held for approximately 4 h, the temperature must be raised to 90 °C to achieve short-time shucking (5–15 s). However, this temperature is close to the rapid denaturation threshold for scallop meat proteins. Even a slight increase in temperature or duration can cause the scallop meat to overcook, thus requiring high operational precision. The above results indicate that the parameters for water bath shucking must be adjusted according to the scallops’ holding time: the longer the holding time, the greater the total heat input required (determined by both water bath temperature and processing time). Simply extending the processing time can easily lead to overcooking of the scallops. Consequently, in practical applications, the water bath temperature must be dynamically adjusted based on the post-harvest holding time of scallops. For scallops held longer, raising the processing temperature reduces the treatment time, balancing shucking efficiency and meat quality. This requirement highlights the necessity of introducing a visual feedback temperature control system in subsequent research, which can automatically adjust the water bath temperature based on the results of doneness detection.

For the convenience of subsequent descriptions, we classified the doneness levels of scallops based on their appearance and quality. The doneness classification criteria (Raw, Medium, Cooked) were established under the professional guidance of food science experts. Based on practical considerations of scallop processing and scallop quality, the experts defined the three doneness levels according to features including color, texture, and mantle contraction. The validity of this classification was further confirmed by non-visual indicators such as ease of adductor muscle detachment, meat firmness, and juice loss. These indicators were evaluated with the involvement of professionals from the scallop processing industry. The classification criteria are summarized in Table 2:

In subsequent studies, “Medium” is chosen as the ideal post-shucking state for scallops, based on a comprehensive assessment of the vibration separation process and final product quality. Specifically, scallops in the “Raw” state, although their adductor muscles may have partially detached after water bath treatment, some degree of adhesion remains. During the subsequent vibration separation process, these adherent samples are subjected to prolonged vibration, resulting in stretching and tearing that causes tissue damage to the scallop meat. This not only affects the product’s visual integrity but also leads to juice loss, thereby compromising product quality and commercial value. In contrast, scallops in the “Medium” state have completely detached adductor muscles from the shell, enabling easy natural separation during vibration processing, while their internal texture and flavor largely retain their fresh state. For scallops in the “Cooked” state, although detachment is also complete, the scallop meat has undergone significant cooking and deterioration, which is inconsistent with our study’s goal of high-quality processing. Therefore, selecting “Medium” as the target state after shucking is the optimal choice that balances high-quality scallop meat while minimizing mechanical damage.

#### 2.1.3. Experiments on the Effects of Adductor Muscle Tension

**Figure 1:**
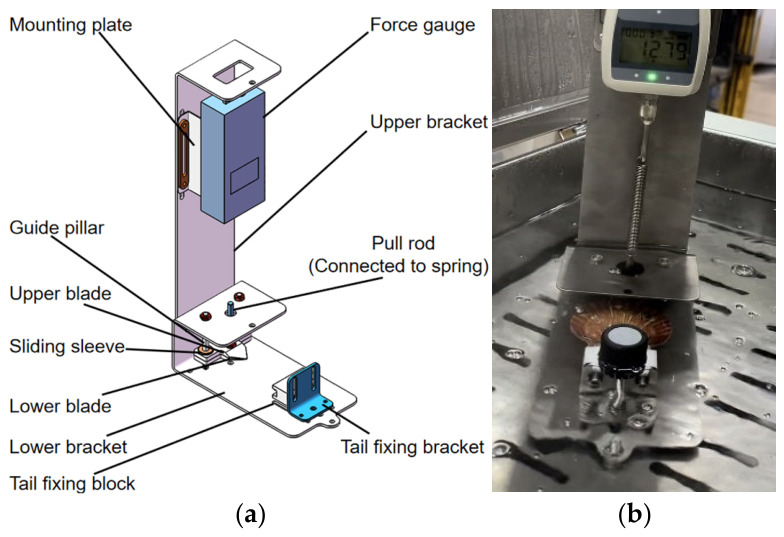
Scallop adductor muscle tension measurement: (**a**) Assembly model of the water environment tension meter for adductor muscle; (**b**) On-site testing.

To further investigate the effects of water bath treatment on the scallop shucking process, tension measurements during the treatment were conducted to quantify the relaxation process of the adductor muscle under thermal treatment, providing additional experimental evidence for shucking parameter selection. To support these measurements, a water-environment tension meter was independently developed for this study, as shown in Figure 1a. By securing the posterior end of the scallop to the tail module, while a small opening at the anterior end is spread open by the tips of the upper and lower blades. The upper blade is connected to a spring, which in turn is connected to a force gauge. Placing the meter in a constant-temperature water bath for real-time measurement of the tension exerted on the adductor muscle during the treatment. As the treatment proceeds, the adductor muscle gradually relaxes. When the scallop’s adductor muscle can no longer withstand the tension applied by the spring, the force gauge reading will undergo a sudden change, indicating that the adductor muscle has ruptured and the shell has opened. By connecting the force gauge to a computer, the real-time tension data can be plotted to obtain a graph showing how the tension exerted on the scallop adductor muscle changes over time at a specific water bath temperature. The on-site test is shown in Figure 1b.

**Figure 2:**
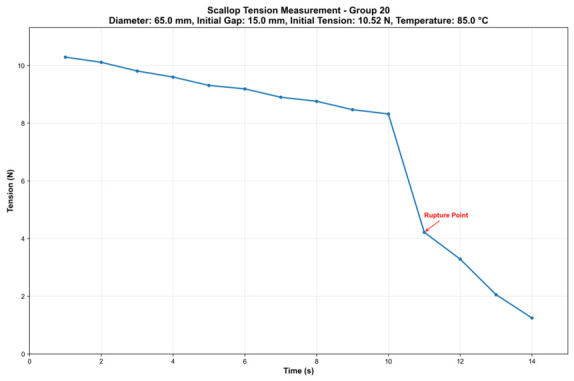
Tension-time curve of the scallop adductor muscle during water bath treatment.

In subsequent experiments, the diameter and initial gape size of the scallop samples were first measured. The samples were then grouped by diameter, and experiments were conducted at different water bath temperatures within each diameter group. The tension exerted on the adductor muscle during water bath treatment was measured in real time. Figure 2 shows a representative tension-time curve of the adductor muscle during water bath treatment.

Experimental results show that the maximum tension the adductor muscle can withstand gradually decreases as water bath treatment time increases. Furthermore, the higher the water bath temperature, the faster the rate of decline in tension. The time to adductor muscle rupture shows a certain correlation with scallop diameter and initial gape size: the larger the scallop diameter and the smaller the initial gape size, the later the adductor muscle ruptures. However, significant individual variations in shucking time were still observed. At the same water bath temperature, the tension of some scallops dropped sharply within a few seconds, with the adductor muscle rapidly reaching its maximum sustainable tension, thereby completing shucking; whereas in other samples, the tension decreased slowly, and the shell remained unopened even after more than 30 s. This indicates that, in addition to water bath temperature, scallop diameter, and initial gape size, individual differences such as physiological state and adductor muscle strength also significantly influence the opening process. Therefore, the subsequent introduction of a visual feedback temperature control system is key to addressing this issue.

### 2.2. Dataset Collection

The scallop doneness image dataset was built using images captured at the image acquisition point after shucking. And to ensure a relatively balanced distribution across the three doneness classes, additional “Cooked” and “Raw” samples were collected by adjusting the processing temperature, as most samples processed within the optimal temperature range naturally fell into the “Medium” category. The image acquisition was concentrated during the bay scallop harvest seasons (November to December 2024 and November to December 2025).

**Figure 3:**
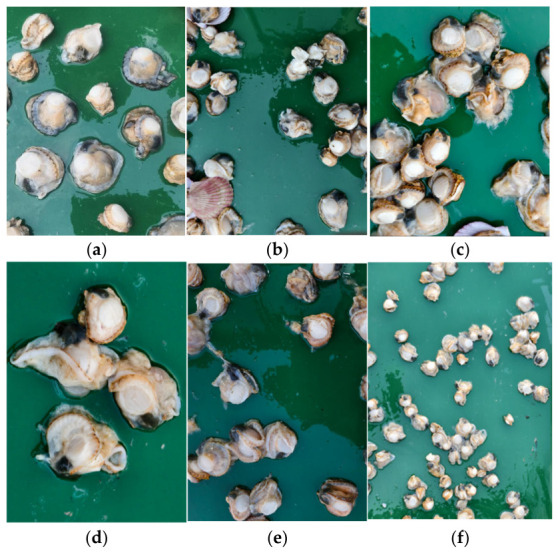
Example images from the dataset: (**a**) Multiple doneness levels; (**b**) Shell interference; (**c**) Dense distribution; (**d**) Close-up view; (**e**) Medium view; (**f**) Distant view.

This scallop doneness image dataset covers scenarios such as complex stacking, varying distributions of doneness levels, and varying scallop counts, reflecting real situations that may occur after shucking. Additionally, to enhance the robustness of the subsequent model, combinations of different lighting conditions, shooting angles, and shooting heights were used during image acquisition. After image acquisition, the images were screened on a PC to remove samples with out-of-focus adductor muscles, ghosting, or other issues that could affect subsequent detection. The final image dataset contained a total of 2310 samples. Example images from the dataset are shown in Figure 3.

Each scallop in the images was annotated using LabelImg for position and doneness level, with categories including Raw, Medium, and Cooked. The dataset was divided into training, validation, and test sets at a ratio of 7:2:1, with each split composed of images from different experimental batches to prevent data leakage, rather than by randomly splitting images from the same batch. And to ensure labeling reliability, a random subset of 200 images was independently annotated by two groups: the food science experts and our research team. Cohen‘s kappa was calculated to measure inter-annotator agreement between the two groups. The resulting kappa value was 0.96, indicating near perfect agreement according to Landis and Koch’s interpretation scale. This high level of agreement confirms the objectivity and reproducibility of our classification criteria. For the remaining images, labels were assigned by our research team following the criteria, with ambiguous cases resolved through consultation with the experts.

**Figure 4:**
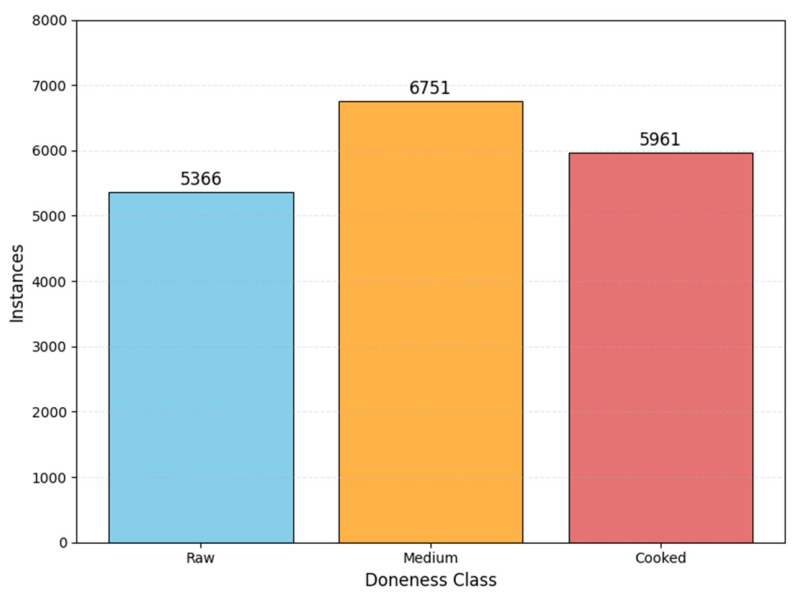
Number of instances in each doneness class.

**Figure 5:**
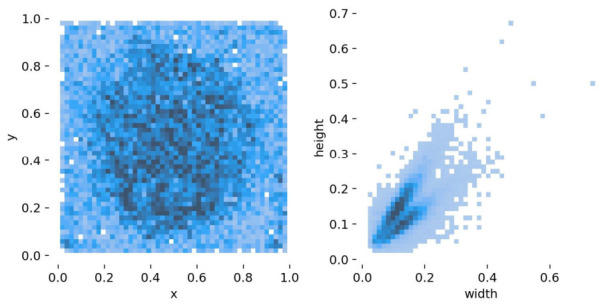
Distribution of bounding box positions and sizes.

This dataset contains a total of 18,078 annotated scallop instances. Figure 4 shows the number of instances in each doneness class. Figure 5 illustrates the distribution of bounding box positions and sizes.

### 2.3. Training Environment and Settings

Model training and inference were conducted on Ubuntu 20.04.6 LTS and CUDA 12.0, using an Intel Xeon-Gold 6248 (2.5 GHz/20-core) processor and an NVIDIA RTX 4090 GPU (48 GB VRAM) as the core computing hardware. The deep learning framework was PyTorch 2.0.0+cu118, and the integrated development environment was Visual Studio Code 1.106.3. The training parameters are set as follows: 300 epochs, batch size of 4, and input image dimensions of 640 × 640. The optimizer used is AdamW (momentum = 0.9), with weight decay set to 0.0001 and an initial learning rate of 0.0001.

### 2.4. Scallop Shucking System with Visual Feedback Temperature Control

To ensure that the doneness of scallops remains within the ideal range after shucking, this study designed and implemented a low-temperature water bath scallop shucking system that integrates the doneness detection model SDD-RT-DETR and a feedback temperature control module.

**Figure 6:**
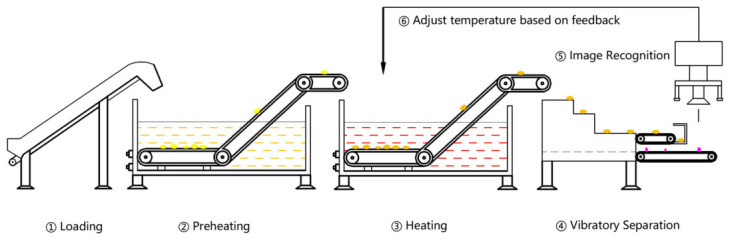
Process flow diagram of low-temperature water bath scallop shucking.

As shown in Figure 6, the system’s process flow is as follows: First, the scallops undergo pretreatment in a preheating tank with a 30 °C water bath to relax the adductor muscle. Subsequently, the pretreated scallops are transported by a conveyor belt into a heating tank. The conveyor belt speed is determined by the length of the heating tank and the optimal shucking time established in the water bath parameters influence experiments described in Section 2.1.2, ensuring that the scallops’ residence time in the heating tank aligns with the optimal shucking time, which effectively inactivates the adductor muscle and breaks its attachment to the shell. The initially detached scallops are then sent to a vibrating screen, where continuous mechanical vibration separates the meat from the shell. On this basis, the visual feedback temperature control module serves as the core of the system, responsible for detecting the doneness of the separated meat and adjusting the heating temperature based on the detection results, thereby forming a closed-loop control.

As the core of the system, the visual feedback temperature control module consists of three highly coordinated units: the vision detection unit, the decision control unit, and the temperature control unit. The vision detection unit is deployed after the vibration separation process and consists of an image acquisition sub-unit and an image processing sub-unit. For image acquisition, this study uses a Sony XCG-CG510C high-performance industrial camera (Sony Corporation, Tokyo, Japan) equipped with an 8 mm fixed focal length industrial lens (F2.8). This camera is equipped with a global shutter sensor, which prevents motion blur caused by object movement on the production line. It connects to the computer via a Gigabit Ethernet interface, supporting stable transmission of high frame rate, high resolution images for subsequent model detection. Working in conjunction with the camera is a linear LED cold light source, which ensures uniform illumination of the scallop surface without shadows in the workshop environment. The image processing sub-unit deploys the high-precision scallop doneness detection model SDD-RT-DETR. The model performs millisecond-level inference on real-time images, accurately identifying and boxing every scallop in the frame, and classifying them into three categories: “Raw”, “Medium”, and “Cooked”. It simultaneously outputs quantity and doneness information to the decision control system. The specific structure and design principles of this model will be detailed in the next chapter.

The decision control unit is based on a high-performance industrial computer running proprietary host monitoring software (self-developed, v1.0). This unit is responsible for system parameter configuration (such as temperature thresholds and batch size), operational status monitoring (live video and temperature curves), and alarm management. Its core decision-making is based on the batch doneness statistics reported by the visual detection unit. It generates the optimal temperature adjustment strategy according to predefined rules and then issues commands to the temperature control unit.

The temperature control unit is key to achieving precise system regulation. The core component of this unit is the BCA series high-precision PID temperature controller. It integrates a self-tuning PID control module, solid-state relay drive circuitry, and an isolated RS485 communication module that supports the Modbus RTU protocol. This unit uses a PT100 platinum resistance temperature sensor to continuously monitor the water bath temperature and employs high-power stainless steel immersion heating rods for heating. Additionally, the unit incorporates circulation pumps to ensure uniform water temperature throughout the bath and prevent localized overheating.

**Figure 7:**
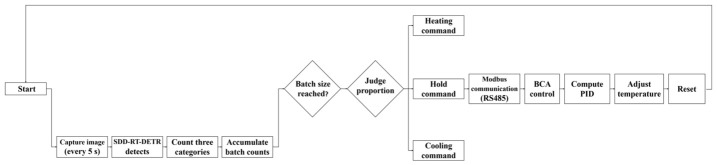
Visual feedback temperature control logic workflow.

Overall, the workflow of the visual feedback temperature control module is a highly automated cyclic process, with the simplified complete control logic illustrated in Figure 7. Every cycle begins with image acquisition: the industrial camera captures images of the production line at a preset interval (5 s), and the scallop doneness detection model SDD-RT-DETR performs real-time inference on the images, automatically adding the identified quantities of each doneness category to the current batch counter. This process continues until the accumulated count reaches the preset batch size (100). Once this quantity is reached, the system immediately enters the decision-making phase: first, it calculates the ratio of “Cooked” and “Raw” scallops in the batch. Next, it compares this ratio against a preset quality threshold. By default, the thresholds for Cooked and Raw are set to 25% and 15%, respectively, to balance practical production feasibility and product quality, minimizing unnecessary adjustments and equipment wear. However, to more rigorously validate the responsiveness of the feedback control strategy, all system tests in this study were conducted with both thresholds set to 10%. If the proportion of “Cooked” exceeds the preset upper threshold, then the current water temperature is judged to be too high, and the system automatically issues a cooling command. Conversely, if the proportion of “Raw” exceeds the threshold, a heating command is issued. The temperature adjustment magnitude is determined based on the extent to which the proportion exceeds the threshold: the larger the deviation, the larger the adjustment step, up to a maximum of 5 °C; smaller deviations result in smaller steps, with a minimum adjustment of 2 °C. The adjustment step size was determined through the earlier experiments and is configurable according to the user‘s requirements.

Then the system enters the execution phase: the decision control unit writes the new temperature setpoint into the designated hold register of the BCA temperature control unit (Xiamen Kudom Electronics Technology Co. Ltd., Xiamen, China) via an isolated RS485 communication cable (Shenyang Shenxingda Cable Co. Ltd., Shenyang, China) using the standard Modbus RTU protocol. Upon receiving the new command, the BCA controller’s internal PID algorithm responds immediately. Based on the deviation between the actual temperature fed back by the PT100 and the new setpoint, it dynamically adjusts the duty cycle of the heating element’s output power, driving the water bath temperature to quickly and smoothly converge toward the new setpoint.

The system aims to maintain the water bath temperature within ±0.3 °C of the setpoint. The image acquisition–inference–decision loop itself takes less than 2 s. The overall system delay, from image acquisition to the start of temperature regulation is less than 5 s. To accelerate heating, multiple high-power heating rods at the bottom work together with circulation pumps to ensure uniform temperature distribution and rapid heat transfer; cooling is also achieved by the pumps. The BCA controller provides a 0.1 °C output resolution, and these hardware constraints define the limits of the system’s adjustment rate and steady-state accuracy. Accordingly, the entire temperature adjust-ment is completed within 2 min, with the preset ± 0.3 °C tolerance predefined in the C# control program. During adjustment, image capture and inference are paused, and the conveyor belt speed is reduced to support thermal stabilization. Notably, temperature adjustments are not triggered frequently; the primary factor influencing the need for adjustment is the post-harvest holding time (freshness) of the scallops, as this directly affects the physiological state of the adductor muscle. This is why the 5 s image acquisition interval was chosen; it allows rapid detection of proportion changes and timely system response once the temperature adjustment is complete. Once the entire temperature adjustment process is complete, the system automatically resets and iterates, clearing the batch counter and immediately initiating a new cycle to achieve continuous process optimization.

**Figure 8:**
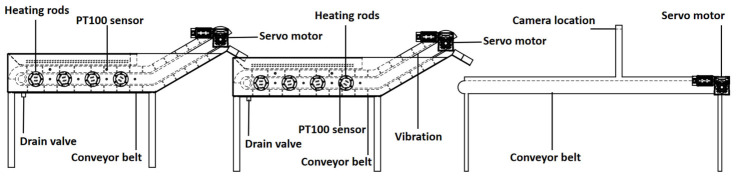
Assembly drawing of the mechanical structure.

**Figure 9:**
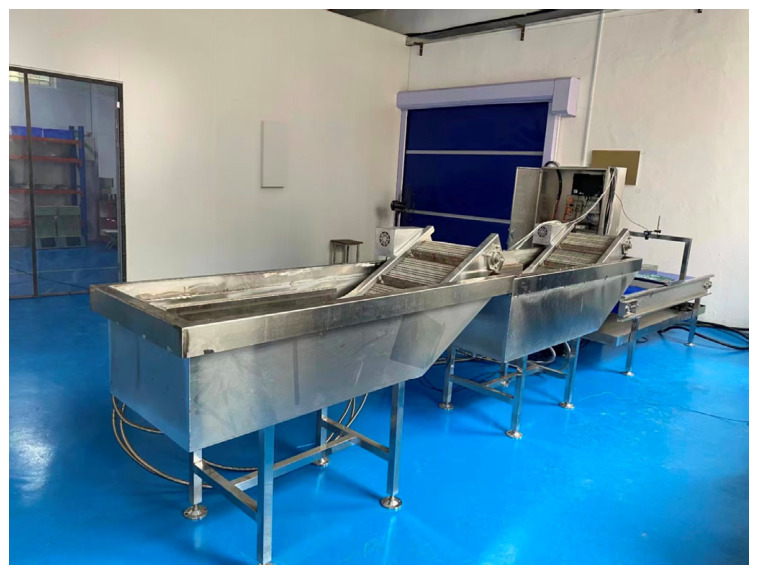
Scallop shucking system in the workshop.

Based on this design, the system can stabilize the doneness of shucked scallops within the ideal range. Regarding settling behavior, the system converges smoothly to the new setpoint without significant overshoot, benefiting from the PID controller and the PWM-based heating control. We also evaluated an alternative control strategy that used detection results to adjust conveyor belt speed. However, this approach proved difficult to control effectively, often shifting the system from undercooked to overcooked extremes, and was therefore not adopted. The system achieves an end-to-end inference latency (including preprocessing, inference, and postprocessing) of less than 50 ms per image, corresponding to a throughput of approximately 20 FPS, which matches the 23 FPS supported by our industrial camera. The entire software system, including the detection model, occupies less than 200 MB of memory, confirming the system’s lightweight nature and suitability for real-time industrial deployment. In terms of power consumption, the heating module (including heating rods, PID controller, and circulation pumps) has a peak power of approximately 26.5 kW, with a dedicated power supply isolated from other components to prevent interference, while the remaining components (industrial computer, camera, etc.) consume about 300 W, totaling roughly 26.8 kW under peak load. In the current industrial setup, the vision detection module is physically separated from the water bath shucking module, so steam and water spray do not affect system operation. Figure 8 presents the assembly drawing of the mechanical structure. Figure 9 presents a photograph of the system in the workshop. Its actual performance will be verified in the subsequent tests in Section 5.2.

## 3. Scallop Doneness Detection Algorithm Design

### 3.1. The SDD-RT-DETR Model Architecture

**Figure 10:**
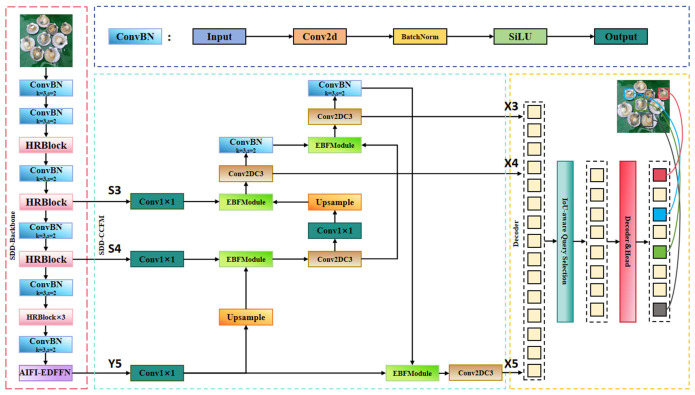
Network architecture of SDD-RT-DETR.

This study introduces SDD-RT-DETR, an end-to-end object detection model designed for the scallop doneness detection task. Its architecture is systematically restructured based on RT-DETR and consists of three components: the backbone network SDD-Backbone, the neck network SDD-CCFM, and the detection head (Decoder). Compared to the baseline model, the improvements of SDD-RT-DETR are as follows: First, the original backbone ResNet18 [37] was replaced with a backbone centered on the self-developed module HierarchicalRepBlock. Second, the original AIFI module is enhanced to form AIFI-EDFFN. Third, in the neck network, the original Concat operation for feature concatenation is replaced by a self-developed EfficientBalanceFusionModule, while the original RepC3 module is replaced by the Converse2DC3 module. Finally, Wise-DIoU is adopted to replace the standard IoU loss function. Among these proposed modules, HierarchicalRepBlock and EfficientBalanceFusionModule are newly proposed, while the remaining modules are adapted from existing designs for the scallop doneness detection task. Their effectiveness is validated through ablation studies in Section 5.1.1, Section 5.1.2 and Section 5.1.3. Figure 10 illustrates the architecture of SDD-RT-DETR.

The model’s inference process is as follows: The input image is uniformly scaled and fed into the SDD-Backbone network. Through four stages of downsampling and feature enhancement via the HierarchicalRepBlock module, outputting feature maps at three scales: S3, S4, and S5. The deepest layer, S5, is further processed by the AIFI-EDFFN to obtain the feature map Y5. Subsequently, S3, S4, and Y5 are concurrently input into the neck network SDD-CCFM, for multi-round cross-scale fusion, outputting the enhanced feature maps X3, X4, and X5. Finally, these feature maps are mapped by the Decoder to obtain the scallop’s bounding box coordinates and doneness class.

### 3.2. Improved Backbone Using HierarchicalRepBlock

To address the feature extraction limitations of the baseline model RT-DETR in the scallop doneness detection task, this study designs a new backbone network, SDD-Backbone, to replace the original ResNet18. Its structure is shown in Figure 10. This backbone is centered on a self-developed multi-path feature extraction module, Hierarch-icalRepBlock, and aims to enhance the model’s ability to capture subtle features such as scallop color and texture that correlate with doneness.

**Figure 11:**
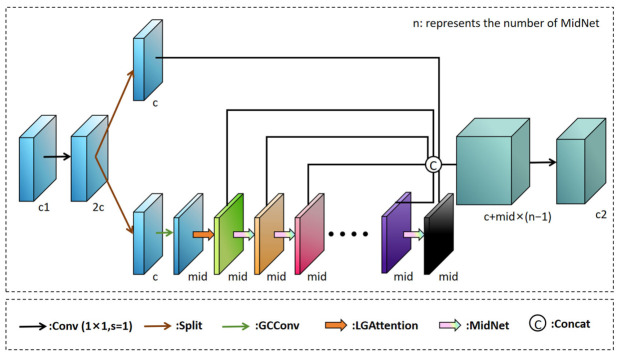
Structure of HierarchicalRepBlock.

The structure of the HierarchicalRepBlock is shown in Figure 11; its processing flow is as follows: the input features first undergo a 1 × 1 convolution to double the number of channels, then split into two parallel branches. One branch is directly output without any further processing to preserve shallow details, while the other branch serves as the main path and proceeds to the deep feature refinement stage.

**Figure 12:**
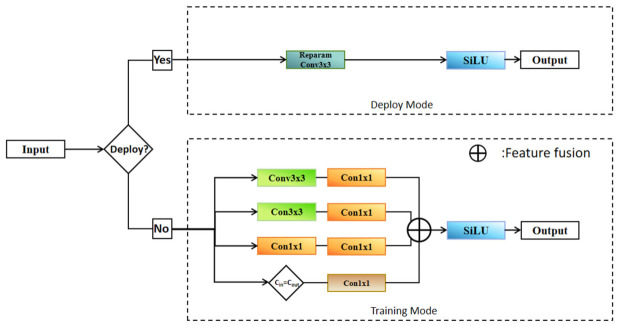
Structure of GCConv.

The main path is first processed by GCConv [38], a multi-branch reparameter convolution. As shown in Figure 12, GCConv employs a multi-path design during the training phase, comprising two independent 3 × 3 convolution branches, one 1 × 1 convolution branch, and an optional residual connection (activated when the number of input and output channels is equal). This multi-branch structure enables the model to fuse features from different receptive fields. During inference, GCConv uses reparameterization to equivalently merge all branches into a single standard 3 × 3 convolution, thereby ensuring inference speed is not compromised. Then the feature maps are fed into the LocalGlobalAttention [39] module. This module divides the feature maps into multiple non-overlapping local patches, compresses the features within each patch, and applies a multi-layer perceptron (MLP) mapping to generate attention weights that reflect the importance of local regions. Simultaneously, the module introduces a learnable global prompt, enabling each local patch to reference global semantic information while computing attention, thereby achieving simultaneous focus on both local features and the overall image. This design allows the module to effectively focus on image regions most relevant to scallop doneness assessment (such as specific color regions on the scallop adductor muscle surface) while suppressing interference from irrelevant background.

After processing by GCConv and LocalGlobalAttention, the features are further refined through intermediate layers (the number of layers is controlled by parameter *n*), each consisting of a 3 × 3 convolution followed by a LocalGlobalAttention module. In each refinement step, features are first extracted by convolution and then enhanced by the attention module to emphasize key regions, ensuring that features relevant to scallop doneness judgment are continuously strengthened instead of diluted during propagation. Subsequently, the output feature maps from all intermediate layers are concatenated with the feature map from the identity branch along the channel dimension, and finally fused via a 1 × 1 convolution to produce a feature map that preserves the rich details of the original maps while possessing strong semantic information.

In the SDD-Backbone network, the HierarchicalRepBlock module is deployed at multiple feature scales (P2/4, P3/8, P4/16, and P5/32), forming a complete feature extraction pipeline that progresses from shallow-level detail capture to high-level semantic enhancement. This design enables the model to simultaneously capture the contour details of scallops (small target detection), fine-grained features (doneness classification), and global morphology (object localization), providing high-quality multi-scale feature maps for the subsequent cross-scale feature fusion network.

### 3.3. Improved AIFI Using EDFFN

**Figure 13:**
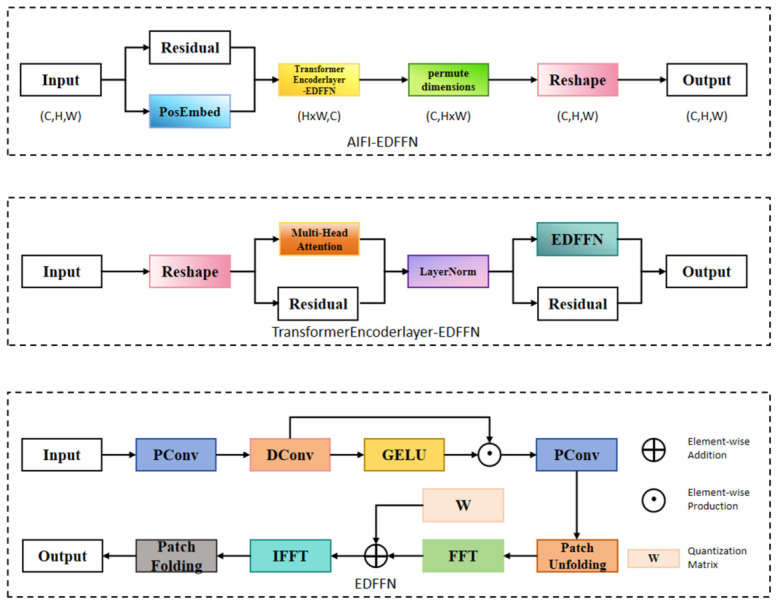
Structure of AIFI-EDFFN.

For the scallop doneness detection task, this study improved AIFI by incorporating the EDFFN [40] module, resulting in AIFI-EDFFN. Its detailed structure is shown in Figure 13.

When the feature maps are input into the AIFI-EDFFN module, it is first split into two branches: one branch adds positional encoding, while the other retains the original features. Both branches are fed in parallel into the TransformerEncoderLayer-EDFFN module. In this module, the feature maps first undergo shape adjustment via Reshape, followed by multi-head self-attention calculations. Subsequently, the feature maps undergo layer normalization before being fed into the EDFFN feedforward network. Unlike the two-layer fully connected network used in the original AIFI module, EDFFN innovatively introduces frequency-domain transformation processing: first, features are transformed from the spatial domain to the frequency domain via a Fourier transform; then, the frequency-domain features are adjusted by learnable weight parameters; and finally, they are reheld in the spatial domain via an inverse Fourier transform.

The AIFI-EDFFN module has unique advantages in the scallop doneness detection task. Its global attention and spatial-frequency dual processing mechanisms effectively address occlusion and overlap between individual scallops, reducing false detections and missed detections in densely distributed scenes. Meanwhile, EDFFN’s multi-layer nonlinear transformation and frequency-domain processing mechanism can capture subtle doneness differences that the original AIFI module struggles to distinguish.

### 3.4. Improved Neck

**Figure 14:**
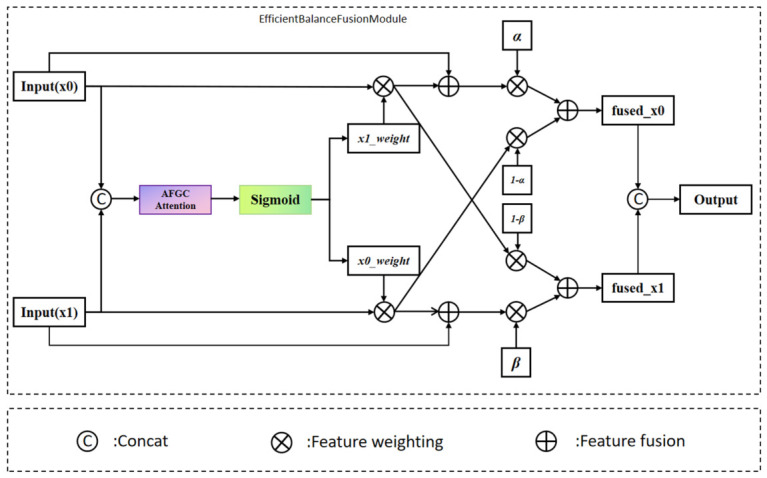
Structure of EfficientBalanceFusionModule.

The improvements to the neck network mainly consist of two parts. First, the channel concatenation operation in the original CCFM is replaced by a self-designed efficient balance fusion module, named EfficientBalanceFusionModule, whose structure is shown in Figure 14.

**Figure 15:**
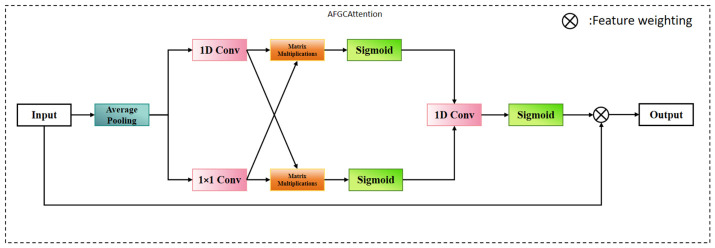
Structure of AFGCAttention module.

Feature maps input into the EfficientBalanceFusionModule are first concatenated along the channel dimension and then fed into the AFGCAttention [41] module for processing, as shown in Figure 15. This attention module first applies average pooling on the input feature maps, then splits them into two separate paths: one path uses 1D convolutions for local interactions along the channel dimension, while the other uses fully connected layers for global channel interactions. Finally, the features from the two branches are first combined via matrix multiplication, then fused, and finally passed through a Sigmoid function to obtain the channel attention weights.

After obtaining the attention weights, the module does not simply perform a weighted sum; instead, it achieves deeper interaction through a learnable bidirectional fusion mechanism. This mechanism introduces a pair of trainable parameters, *α* and *β*, constrained within the interval (0, 1) via the Sigmoid function. During training, these parameters dynamically learn the optimal fusion ratio between the two input features. The core fusion formula is as follows:

*fused_x*0 = *α* × *x*0 × (1 + *x*1*_weight*) + (1 − *α*) × *x*1 × *x*0*_weight* (1)

*fused_x*1 = *β* × *x*1 × (1 + *x*0*_weight*) + (1 − *β*) × *x*0 × *x*1*_weight* (2)

In this formula, the feature *fused_x*0 is determined not only by itself and the complementary weight from *x*1 (*x*1*_weight*), but also incorporates complementary information from *x*1 that has been filtered through the weight of *x*0 (*x*0*_weight*). Similarly, the feature *fused_x*1 follows the same principle. This design ensures that the fusion is bidirectional and meaningful. For example, the semantic information from high-level features, such as “this region may be a cooked scallop”, can serve as a signal to enhance the corresponding color and texture details in low-level features. Conversely, the spatial details in low-level features can provide precise localization correction for high-level features, compensating for the blurred localization of small target scallop localization caused by downsampling.

**Figure 16:**
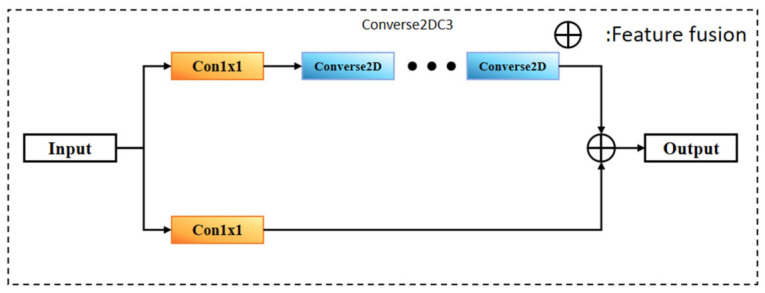
Structure of Converse2DC3.

On the other hand, to enhance the feature extraction capability of the neck network, this study replaces the RepC3 module, which is built on RepConv (re-parameterized convolution), with the Converse2DC3 module, which is built on Converse2D [42] module (frequency-domain processing convolution). Its structure is shown in Figure 16.

Converse2DC3 is deployed after EfficientBalanceFusionModule, as shown in Figure 7. It further extracts and enhances the fused features. For the scallop doneness detection task, its main roles are as follows: In the upsampling path, this module can effectively recover and enhance the high-frequency details lost in high-level features due to downsampling. In the downsampling path, this module helps low-level features incorporate necessary spatial details while retaining high semantic information. Additionally, the frequency-domain processing of the Converse2DC3 module makes the model more robust to illumination variations, effectively reducing classification errors caused by environmental factors.

### 3.5. Improved Loss Function

The improved loss function, Wise-DIoU, combines the dynamic weighting of Wise-IoU [43] with the *DIoU* [44] calculation method. Traditional IoU treats all samples equally. However, in this scallop doneness detection task, samples of different difficulty levels contribute significantly differently to model training: Easy samples (such as isolated, large-sized scallop targets) typically achieve high IoU values quickly, but their gradient contribution is limited; hard samples (such as severely occluded or highly overlapping scallop targets) often have lower IoU values, poor matching quality, and are difficult to optimize; whereas samples of moderate difficulty (with IoU between 0.3 and 0.7) typically offer the greatest learning value. Therefore, Wise-IoU uses a dynamically updated average IoU value (iou_mean) as a reference benchmark and defines the relative IoU ratio of a sample (β = L_iou_/L_iou_mean_) as an outlier degree to describe the quality of the anchor boxes. Wise-IoU uses the outlier degree β to define a weight function: β/(δ × α^β−δ^), which is used to dynamically adjust the gradient gain of the sample, where the hyperparameters α = 1.7 and δ = 2.7 are set according to this task. In this function, a smaller gradient gain is assigned when β deviates far from 1, corresponding to easy (small β) or hard (large β) samples, whereas a larger gradient gain is assigned when β is close to 1, corresponding to samples of moderate difficulty. This design enables the model to focus more on informative samples during training, thereby effectively improving training efficiency.

Additionally, this loss function incorporates the *DIoU* loss. The *DIoU* loss formula is:

(3) $$L_{DIoU}=1-IoU+\frac{\rho^{2}(b,b^{gt})}{c^{2}}$$

where *b* and *b^gt^* represent the center points of the predicted bounding box and the ground truth bounding box, respectively; *ρ* is the Euclidean distance between the two center points; and *c* is the diagonal length of the smallest bounding rectangle that contains both boxes. This design introduces the distance between the two center points as an additional penalty term, forcing the model to align the center points of the predicted and ground truth boxes while optimizing the overlap area. For scallop targets, which are roughly circular or elliptical, center alignment ensures that the bounding box can more completely cover the entire scallop. Therefore, *DIoU* is well suited for this detection task. Additionally, Wise-IoU supports various other IoU variants, such as GIoU [45], CIoU [44], EIoU [46], and SIoU [47], to accommodate different requirements.

## 4. Evaluation Indicators

This study adopts two types of metrics to evaluate model detection performance and model complexity. The detection performance metrics include precision (P), recall (R), F1 score, and mean Average Precision (mAP). The complexity metrics include the number of parameters and computational cost (GFLOPs). The formulas for the detection performance metrics are as follows:

(4) $$\;Precision=\frac{TP}{TP+FP}$$

In the formula, true positive (*TP*) represents the number of samples correctly classified as positive, false positive (*FP*) represents the number of samples incorrectly classified as positive, and precision is the proportion of True Positives among all predictions classified as positive. In this task, for each doneness category, precision is defined as the proportion of scallops that actually belong to that category among all scallops predicted by the model to belong to that category.

(5) $$Recall=\frac{TP}{TP+FN}$$

In the formula, false negative (*FN*) represents the number of samples incorrectly predicted as negative, and recall is the proportion of samples correctly predicted as positive among those that are actually positive. In this task, for each doneness category, recall is defined as the proportion of scallops actually belonging to a given category that are correctly predicted as that category by the model.

(6) $$F1=\left(\frac{2}{Recall^{-1}+Precision^{-1}}\right)=2\frac{Precision\times Recall}{Precision+Recall}\;$$

In the formula, the *F*1 score represents the harmonic mean of precision and recall. The closer the value is to 1, the better the model detection performance.

(7) $$AP=\int_{0}^{1}P(r)dr$$

In the formula, *AP* represents the area under the precision-recall curve for a single doneness category, which reflects the precision and recall of that category under different confidence thresholds.

(8) $$mAP=\frac{\sum_{j=1}^{S}AP(j)}{S}$$

In this formula, *mAP* averages the APs across all categories, comprehensively considering the precision and recall of each category. A higher *mAP* indicates better detection performance of the model. mAP@50 (mean average precision at IoU threshold of 0.5) reflects the model’s performance under a lenient standard, while mAP@50:95 (mean average precision over IoU thresholds from 0.5 to 0.95) more strictly evaluates the model’s detection capability.

## 5. Results and Analysis

### 5.1. Comprehensive Analysis of SDD-RT-DETR

SDD-RT-DETR was trained and evaluated on the self-built scallop doneness image dataset. Table 3. Doneness detection performance of the SDD-RT-DETR model presents the model’s precision, recall, mAP@50, and mAP@50:95 for this task. For specific categories, the model achieved the best detection performance on raw scallops, with precision, recall, and mAP@50 all exceeding 97%. The model also performed well on cooked scallops, achieving 95.9% mAP@50. For medium scallops, which constitute the largest number of samples, the detection accuracy is slightly lower than the other two categories, yet the model maintains a high precision of 93.8% and a recall of 91.6%. Additionally, the model’s average inference time per image is 49.3 ms, meeting the real-time detection requirements of the production lines.

**Figure 17:**
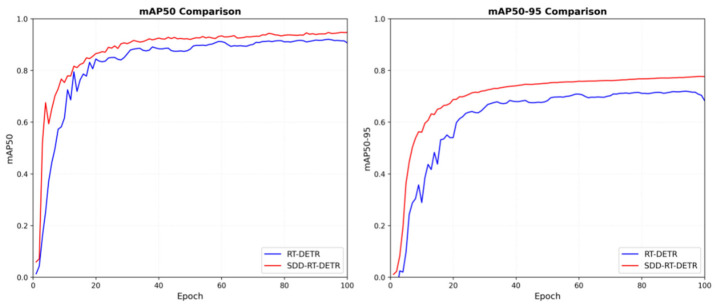
The mAP curves during the first 100 training epochs.

Figure 17 shows a comparison of the mAP@50 and mAP@50:95 curves for the baseline model and SDD-RT-DETR during the first 100 epochs of training. As shown in the figure, both metrics of the improved model consistently outperform those of the baseline model, indicating that the proposed SDD-RT-DETR has a clear advantage in the scallop doneness detection task.

To further evaluate the stability of the proposed model, we repeated the training of SDD-RT-DETR and RT-DETR with 5 different random seeds (1, 3, 42, 99, 123,). For SDD-RT-DETR, the mean mAP@50:95 was 78.360%, with a standard deviation of 0.114%. For RT-DETR, the mean mAP@50:95 was 71.240%, with a standard deviation of 0.260%, confirming that the performance improvements are consistent across different random seeds. And the standard deviations indicate that the observed performance gaps are not due to random variation.

#### 5.1.1. Backbone Architecture Ablation Study

To validate the effectiveness of the proposed SDD-Backbone network, six backbone variants were designed and compared. These variants include lightweight, uniform, heavily stacked, shallow-dense, and deep-dense configurations to investigate the impact of module quantity and distribution on detection performance. The detailed architectures of all variants are shown in Table 4, with all other structures and training settings kept identical.

The experimental results are presented in Table 5. Among all tested backbones, SDD-Backbone achieves the best balance between detection accuracy and model complexity, confirming the effectiveness of the progressive module distribution strategy in HierarchicalRepBlock for capturing both spatial details and semantic features.

#### 5.1.2. Attention Mechanism Ablation Study

To evaluate the proposed AIFI-EDFFN module, we compared it against four widely used attention mechanisms integrated into the same AIFI module, including DAttention (Deformable Attention) [48], ECA (Efficient Channel Attention) [49], PSA (Pyramid Split Attention) [50], and the original AIFI module. All experiments replaced only the attention module while keeping other structures and training settings identical to ensure fair comparison. The results are shown in Table 6.

As shown in table above, AIFI-EDFFN consistently outperforms the other attention variants in both mAP@0.5 and mAP@0.5:0.95. While these alternative mechanisms perform well in their original tasks, they are less effective for scallop doneness detection, confirming the advantage of the frequency-domain processing in EDFFN for capturing fine-grained color and texture features relevant to this task.

#### 5.1.3. Neck Network Ablation Study

To evaluate the proposed SDD-CCFM network, this work compared it against four mainstream feature fusion networks, including SlimNeck [51], HSPAN [52], BiFPN [53], and RepGDNeck [54]. All experiments replaced only the neck network while keeping other structures and training settings identical to ensure fair comparison. The results are shown in Table 7.

As shown in table above, SDD-CCFM achieves the highest detection accuracy among all compared modules, with relatively low parameter count and computational cost, demonstrating the advantage of its bidirectional fusion and frequency-domain processing.

#### 5.1.4. Ablation Study

In this ablation study, we sequentially introduced four improved modules on the baseline RT-DETR model: SDD-Backbone, AIFI-EDFFN, SDD-CCFM, and Wise-DIoU, and compared them in terms of precision, recall, mAP, parameters, and GFLOPs to evaluate the contribution of each improved module. The experimental results are shown in Table 8.

As shown in the table, with the introduction of improved modules, the model significantly reduces complexity while enhancing detection performance. Specifically, after adopting the SDD-Backbone network centered on HierarchicalRepBlock, the number of parameters was significantly reduced to 16.95 M, and mAP@50 improved from 91.9% to 93.2%. And the introduction of the AIFI-EDFFN module further improves mAP@50 from 93.2% to 93.8% with only a minimal increase in parameters. The introduction of SDD- CCFM increases the number of parameters only slightly but improves precision by 3.6%. Finally, by replacing the original loss function with Wise-DIoU, the complete model achieved a precision of 95.5%, a recall of 93.6%, a mAP@50 of 96.1%, and a mAP@50:95 of 78.4%, with only 17.20 M parameters and 8.30 GFLOPs of computational cost. The above results fully validate the effectiveness of the proposed improvements in this study.

#### 5.1.5. Comparison of Different Detection Models

To comprehensively evaluate the performance of SDD-RT-DETR in this task, we conducted systematic model comparison experiments, selecting current mainstream detection models (Faster R-CNN, YOLOv5, YOLOv8, YOLOv10, YOLOv11, YOLOv12) alongside RT-DETR and proposed SDD-RT-DETR. To ensure fair comparison, all models were trained from scratch on the same dataset using identical training settings: 300 epochs, batch size 4, AdamW optimizer, learning rate 0.0001, weight decay 0.0001, seed 42, input size 640 × 640, and the same data augmentation strategies. Experimental results shown in Table 9. It can be seen that SDD-RT-DETR outperforms all comparison models in terms of detection accuracy. Compared to the baseline model RT-DETR, the mAP@50 increased by 4.2%; compared to YOLOv10, the best performing model among the comparison models, it maintains a 5.6% advantage in mAP@50. Additionally, both the model size and computational cost of SDD-RT-DETR are lower than those of the other comparison models.

**Figure 18:**
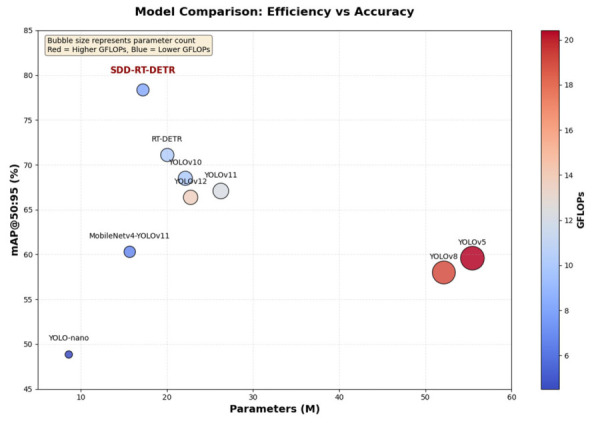
Three-dimensional comparison of parameters, computational cost, and mAP@50:95.

Figure 18 shows a three-dimensional comparison of parameter count, computational cost, and mAP@50:95. It intuitively illustrates the performance distribution of each model. The circular marker of SDD-RT-DETR has a blue color (indicating low GFLOPs) and is located in the optimal efficiency region with a competitive parameter count and the highest mAP@50:95. Thus, this model achieves high-precision detection while meeting the practical requirements of lightweight design for industrial applications.

#### 5.1.6. Visual Analysis

To comprehensively evaluate the detection performance of SDD-RT-DETR in real-world complex production environments, this study designed three representative and challenging scenarios for testing and comparison: (1) Stacked and mutually occluded scallops: this tests the model’s capability to distinguish dense targets. (2) Fine-grained doneness classification: this examines the model’s sensitivity to subtle features related to doneness. (3) Small target detection: this assesses the model’s capability to detect small-sized scallops. All test images were collected separately for each scenario and were not used in model training or validation.

**Figure 19:**
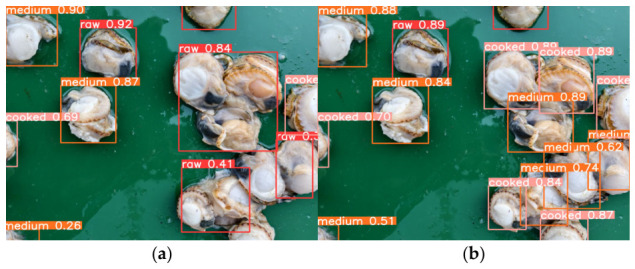
Comparison of dense target detection: (**a**) Baseline; (**b**) SDD-RT-DETR.

Figure 19 shows a comparison of dense target detection. In the left image, the baseline model RT-DETR has obvious defects when detecting densely distributed scallop samples. It tends to detect multiple adjacent scallop targets as a single one and also suffers from many missed detections. In contrast, the right image shows that SDD-RT-DETR effectively solves these problems. It accurately detects and localizes every individual scallop. This improvement is mainly attributed to two factors. First, the improved backbone centered on HierarchicalRepBlock retains richer local feature details. Second, the combination of the AIFI-EDFFN module’s spatial-frequency dual processing mechanism and the dynamic weighted loss function Wise-DIoU significantly enhances the model’s capability to distinguish densely distributed targets.

**Figure 20:**
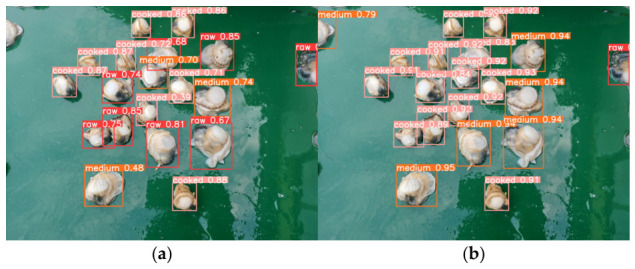
Comparison of scallop doneness classification: (**a**) Baseline; (**b**) SDD-RT-DETR.

Figure 20 shows a comparison of doneness classification. In great difficulty scenes where doneness differences among individual scallops are subtle, the baseline model exhibits significant classification confusion. For example, as shown in the left image, it misclassifies “Cooked” as “Medium” or “Raw”. In contrast, SDD-RT-DETR in the right image accurately classifies all scallops. This result strongly demonstrates that the improved backbone centered on HierarchicalRepBlock provides rich doneness-related features, combined with the frequency-domain processing mechanisms of AIFI-EDFFN and Converse2DC3, enabling the model to capture color and texture features associated with doneness more accurately, thereby achieving more precise doneness classification.

**Figure 21:**
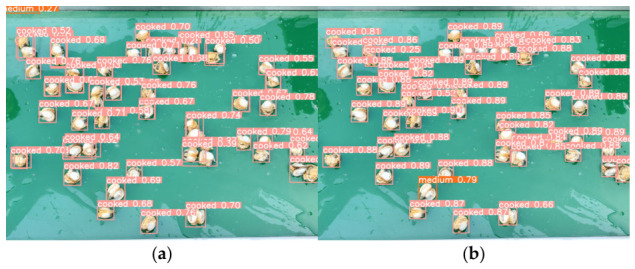
Comparison of small target detection: (**a**) Baseline; (**b**) SDD-RT-DETR.

Figure 21 shows a comparison of small target detection. In scenes where scallops are relatively small, the baseline model in the left image missed and misclassified many targets. In contrast, SDD-RT-DETR in the right image effectively solves this problem and significantly improves detection accuracy for small targets. This improvement mainly comes from the targeted design of the model structure: on one hand, the reconstructed backbone effectively preserves more information of small targets. On the other hand, the neck network SDD-CCFM, through the bidirectional fusion mechanism of EfficientBalanceFusionModule, alleviates the loss of small target feature information in deep networks, thereby greatly enhancing the model’s small target detection capability.

**Figure 22:**
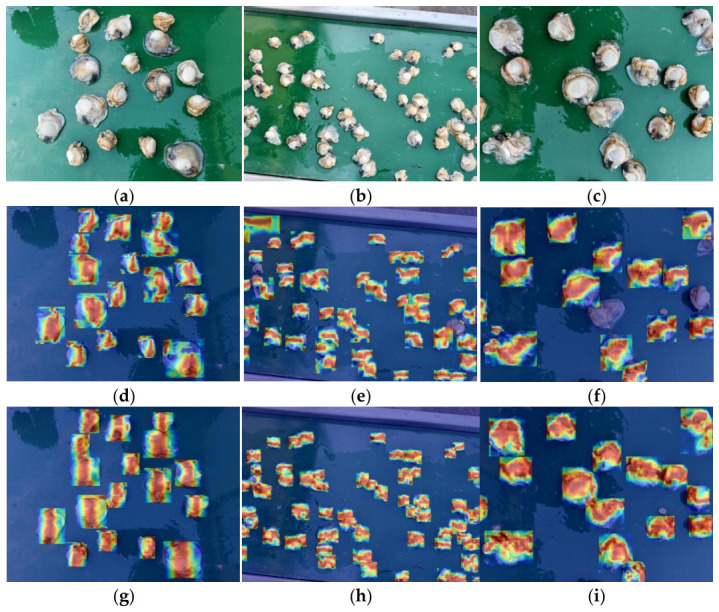
Example heatmaps generated by GradCAM++: (**a**–**c**) Original images; (**d**–**f**) RT-DETR results; (**g**–**i**) SDD-RT-DETR results.

To visually illustrate the model’s focus regions in the scallop doneness detection task, this study performed heatmap visualization on the baseline model and SDD-RT-DETR using GradCAM++. As shown in Figure 22, regions in redder colors indicate higher attention. In comparison, the baseline model does not fully focus on the main part of the scallops. In contrast, SDD-RT-DETR concentrates more on the target regions and effectively suppresses attention to non-target areas, directly validating the effectiveness of the improvements.

In summary, from evaluation metrics to challenging scenarios, the experimental results comprehensively and consistently confirm the superior performance of SDD-RT-DETR in the scallop doneness detection task. This model effectively addresses three critical challenges that frequently occur in real production: distinguishing densely distributed targets, differentiating subtle doneness variations, and reducing false and missed detections of small targets. Thus, it provides a reliable deep learning-based detection solution for both the proposed system and the scallop processing industry.

### 5.2. Visual Feedback Temperature Control System Testing

To verify the practical performance of the proposed shucking system, we have conducted tests under various conditions, including on different days, under different lighting environments (indoor, outdoor sunny, and outdoor cloudy), as well as on scallop batches of varying sizes. The test results confirmed the system‘s effectiveness and robustness under various scenarios. To further evaluate the system under real-world production conditions and compare it with conventional methods, we conducted consecutive batch tests in a representative production setting, these tests were conducted during the local scallop peak harvest season (mid-to-late November) in an actual workshop environment, covering scallops of varying sizes from different batches to ensure representativeness. For these tests, a control group and an experimental group were set up for comparison. Both groups used bay scallops that had been held for 2–4 h after harvesting as test samples, with 50 scallops per batch. The control group used a fixed-temperature water bath shucking with parameters determined from the experimental results in Section 2.1.2 (constant temperature of 85 °C). The experimental group used the proposed low-temperature water bath shucking system, integrating a visual feedback temperature control module with an initial temperature of 85 °C; the thresholds for both Cooked and Raw proportions were set to 10%. This relatively low threshold was chosen to ensure tighter quality control and a higher proportion of properly cooked scallops, at the cost of more frequent temperature adjustments. Once activated, the system operated fully automatically without manual intervention.

The experiment used shucking rate (proportion of scallops successfully detached with intact meat relative to total input) and properly cooked rate (proportion of scallops with doneness level “Medium” among those successfully opened) as core evaluation metrics. The temperature control accuracy and temperature adjustment time of the experimental group system were also recorded. Both groups underwent 20 consecutive batches of testing. The results showed that the experimental group achieved an overall shucking rate of 96.6%, with the lowest batch at 92.0% and the highest at 100%, significantly outperforming the control group’s range of 68.0% to 100% (overall 84.2%). This indicates that the visual feedback temperature control method effectively resolves the adhesion problem between the adductor muscle and the shell, ensuring complete separation of the scallops during the subsequent vibration process. Regarding the properly cooked rate, after the experimental group system stabilized, the properly cooked rate for each batch remained between 86.0% and 94.0%, with an overall rate of 88.5%, significantly higher than the control group’s 74.5%. The control group’s system, unable to adapt to variations in raw material holding time, resulted in a properly cooked rate fluctuating between 62.0% and 92.0%.

To statistically validate the performance improvement, we conducted a paired *t*-test to compare the feedback control method and the fixed-parameter method. For each of the 20 batch runs, the shucking rate and properly cooked rate were paired by batch. The null hypothesis was that there is no significant difference between the two methods, with a significance level set at α = 0.05. For the shucking rate, the feedback control method achieved a mean of 96.6% with a standard deviation of 3.2%, compared to 84.2% with a standard deviation of 12.0% for the fixed-parameter method (*p* < 0.001). For the properly cooked rate, the feedback control system achieved a mean of 88.5% with a standard deviation of 3.9%, compared to 74.5% with a standard deviation of 10.9% for the fixed-parameter method (*p* < 0.001). Since the *p*-values are below the α threshold, the null hypothesis is rejected, confirming that the proposed feedback control system significantly outperforms the fixed-parameter method. Furthermore, during the entire test period, the experimental system operated stably, processing over 1000 scallops without any system failures.

**Figure 23:**
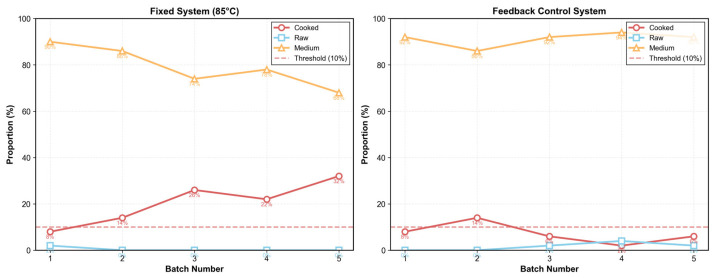
Category-ratio trajectories.

**Figure 24:**
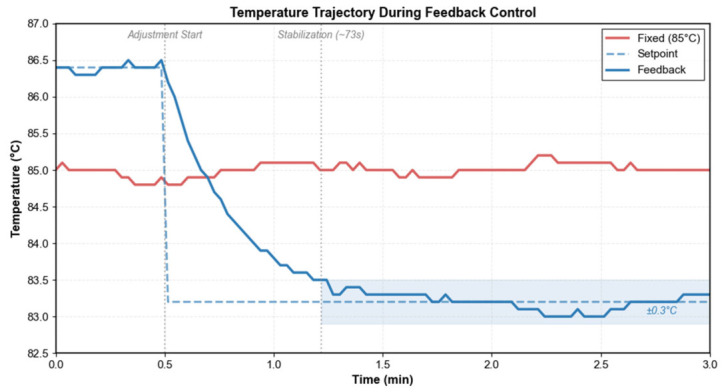
Temperature trajectories.

To further illustrate the dynamic response of the feedback control system, we selected five representative batches from the consecutive tests for detailed analysis. As shown in Figure 23, both systems exceeded the 10% cooked proportion threshold in the second batch. However, the feedback control system responded immediately by lowering the setpoint from 86.4 °C to 83.2 °C, and as shown in Figure 24, the temperature stabilized at 83.2 °C ± 0.3 °C within approximately 73 s without significant overshoot or undershoot (minor oscillations due to measurement noise and thermal inertia are normal). As a result, the feedback system maintained the medium proportion consistently between 86% and 94% across the five batches, with the cooked proportion remaining below the threshold after adjustment. In contrast, the fixed-parameter system (85 °C) could not adjust and thus exhibited large fluctuations in Cooked (8–32%) and Medium (68–90%) proportions. These results confirm that the proposed feedback control strategy effectively stabilizes the doneness distribution across batches.

The above results indicate that the shucking system designed in this study can dynamically adjust the water bath temperature based on the real-time doneness status of the scallops, maintaining a high shucking rate while stabilizing the properly cooked rate within the ideal range. Compared to the traditional fixed-parameter shucking method, this system demonstrates superior performance in both shucking efficiency and quality control, meeting industrial demands.

**Figure 25:**
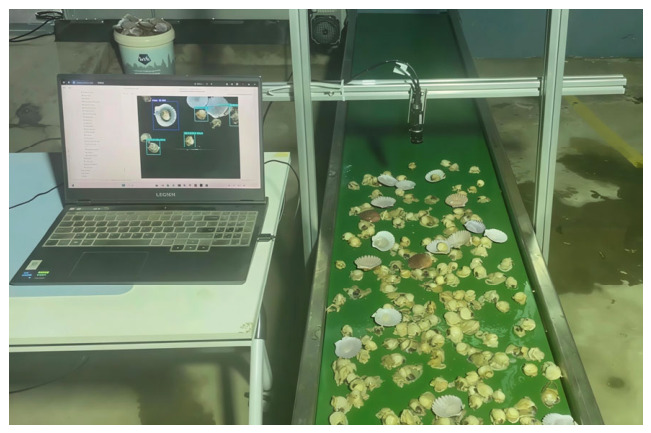
System field testing.

Field testing is shown in Figure 25.

## 6. Conclusions

To address the long-standing issues of low automation and inconsistent quality in the scallop shucking process, this study first conducted comparative experiments to establish low-temperature water bath shucking as the baseline method. The effects of water bath parameters on shucking performance and doneness were investigated and quantified, establishing a parameter baseline for this method. Based on this, this study constructed an image dataset of scallops covering various levels of doneness, providing support for subsequent model training and validation. For the scallop doneness detection task, this study systematically redesigned the structure of RT-DETR, resulting in the scallop doneness detection model SDD-RT-DETR, which serves as the technical core of the entire system. SDD-RT-DETR achieved precision, recall, mAP50, and mAP50-95 of 95.5%, 93.6%, 96.1%, and 78.4%, respectively, representing improvements of 4.7%, 3.2%, 4.2%, and 7.3% over the baseline model. Additionally, computational complexity was reduced by 18.9%, and the number of parameters was reduced by 14.1%. This model can not only be deployed in this shucking system but also extended to scallop doneness detection and sorting. Also, its structure can serve as a reference for doneness recognition tasks of other shellfish, such as abalone and oyster. Finally, this study constructed a low-temperature water bath shucking system integrating the scallop doneness detection model SDD-RT-DETR and a feedback temperature control module. Test results validated the system’s effectiveness in improving automation and stabilizing product quality. In particular, it overcomes the key quality issue of “overcooking”, which is common in existing automated scallop shucking methods. We acknowledge that no suitable public dataset is currently available for benchmarking due to the highly specific nature of this task, and we will continue to actively search for and monitor any relevant public datasets that may emerge in the future. Nonetheless, this study provides an intelligent upgrade solution that integrates an advanced computer vision model and control technologies for scallop processing and the broader food processing industry.

## Figures and Tables

**Table 1:** Shucking time and sensory evaluation at different temperatures (holding time < 2 h).

Water Bath Temperature (°C)	Shucking Time(s)	Sensory Evaluation of Shucking
75	>30	No doneness of the adductor muscle is observed, but most samples remain adhered to the shell.
85	5–20	The surface of the adductor muscle showed slight cooking, durations exceeding 15 s, mantle contraction occurs; very few samples remain adhered to the shell.
95	2–10	Overall doneness is substantial; most samples exhibit noticeable contraction of the mantle, and it is difficult to control the processing time.

Note: The shucking times in the table exclude a few outlier samples and reflect the true distribution of over 90% of the samples.

**Table 2:** Doneness level classification for scallops.

Doneness Level	Visual Appearance	Key Attributes
Raw	No signs of doneness on the adductor muscle surface; mantle shows no contraction; surface appears translucent and moist	Adductor muscle tends to adhere to the shell; firm texture; no color change
Medium	Adductor muscle surface shows slight doneness; mantle has slightly contracted; surface becomes slightly opaque	Adductor muscle detaches naturally from the shell; meat remains elastic; internal texture and flavor remain fresh
Cooked	Adductor muscle is fully cooked; mantle is fully contracted; surface appears opaque and dryish	Adductor muscle detaches completely from the shell; meat becomes firm and tough; visible color and texture changes

**Table 3:** Doneness detection performance of the SDD-RT-DETR model.

Class	Precision (%)	Recall (%)	mAP@50 (%)	mAP@50:95 (%)
All	95.5	93.6	96.1	78.4
Raw	97.7	97.4	97.5	85.0
Cooked	95.1	91.8	95.9	76.3
Medium	93.8	91.6	94.9	73.9

**Table 4:** Comparison of detailed architectures for different backbones.

Variant	P1/2	P2/4	P3/8	P4/16	P5/32
A: Lightweight variant	Conv [64, 3, 2]	Conv [128, 3, 2] HRBlock [128, 0.5] × 1	Conv [256, 3, 2] HRBlock [256, 0.5] × 1	Conv [384, 3, 2] HRBlock [384, 1.0] × 1	Conv [384, 3, 2] HRBlock [384, 1.0] × 1
SDD-backbone	Conv [64, 3, 2]	Conv [128, 3, 2] HRBlock [128, 0.5] × 1	Conv [256, 3, 2] HRBlock [256, 0.5] × 1	Conv [384, 3, 2] HRBlock [384, 1.0] × 1	Conv [384, 3, 2] HRBlock [384, 1.0] × 3
B: Uniformly Distributed Variant	Conv [64, 3, 2]	Conv [128, 3, 2] HRBlock [128, 0.5] × 2	Conv [256, 3, 2] HRBlock [256, 0.75] × 2	Conv [384, 3, 2] HRBlock [384, 1.0] × 2	Conv [384, 3, 2] HRBlock [384, 1.0] × 2
C: Heavily stacked variant	Conv [64, 3, 2] HRBlock [64, 0.25] × 1	Conv [128, 3, 2] HRBlock [128, 0.5] × 2	Conv [256, 3, 2] HRBlock [256, 0.75] × 3	Conv [384, 3, 2] HRBlock [384, 1.0] × 4	Conv [384, 3, 2] HRBlock [384, 1.0] × 5
D: Shallow-Dense Variant	Conv [64, 3, 2]	Conv [128, 3, 2] HRBlock [128, 0.5] × 3	Conv [256, 3, 2] HRBlock [256, 0.75] × 2	Conv [384, 3, 2] HRBlock [384, 1.0] × 1	Conv [384, 3, 2] HRBlock [384, 1.0] × 1
E: Deep-Dense Variant	Conv [64, 3, 2]	Conv [128, 3, 2] HRBlock [128, 0.5] × 1	Conv [256, 3, 2] HRBlock [256, 0.75] × 1	Conv [384, 3, 2] HRBlock [384, 1.0] × 2	Conv [384, 3, 2] HRBlock [384, 1.0] × 4

**Table 5:** Experimental results comparing different backbones.

Backbone Variant	Params (M)	GFLOPs	mAP@0.5	mAP@0.5:0.95
A: Lightweight variant	−32.8%	−22.1%	−3.2%	−4.1%
SDD-Backbone (Ours)	Benchmark	Benchmark	Benchmark	Benchmark
B: Uniform Distribution Variant	+4.9%	+21.7%	−0.3%	−0.7%
C: Heavily stacked Variant	+90.6%	+87.7%	+0.2%	+0.4%
D: Shallow-Dense Variant	−26.9%	+10.7%	−1.5%	−1.8%
E: Deep-Dense Variant	+49.2%	+33.2%	−0.2%	+0.1%

**Table 6:** Performance comparison of different attention modules.

Model Variants	Attention Mechanism Type	Feedforward Network	Position Encoding	mAP@0.5	mAP@0.5:0.95
AIFI-EDFFN (Ours)	Multi-head Self-Attention	EDFFN	2D Sine Encoding	Benchmark	Benchmark
AIFI	Multi-head self-attention	Standard Linear FFN	2D Sine Encoding	−0.7%	−1.1%
AIFI-DAttention	Deformable Attention	Convolutional FFN	None	−2.1%	−3.4%
AIFI-ECA	Efficient Channel Attention	Convolutional FFN	None	−1.9%	−2.5%
AIFI-PSA	Pyramid Split Attention	Convolutional FFN	None	−1.1%	−1.6%

**Table 7:** Performance comparison of different feature fusion modules.

Model Name	mAP@0.5 (%)	Number of Parameters (M)	Computational Cost (GFLOPs)
SDD-CCFM (Ours)	Benchmark	0.366	1.18
SlimNeck	−3.9%	0.217	0.74
HSPAN	−0.7%	0.369	1.04
BiFPN	−1.5%	0.332	1.69
RepGDNeck	−0.2%	3.264	3.53

**Table 8:** Results of the ablation study.

Methods	SDD- Backbone	AIFI_ EDFFN	SDD- CCFM	Wise- DIoU	Precision (%)	Recall (%)	mAP@50 (%)	mAP@50:95 (%)	Params	GFLOPs
①	-	-	-	-	90.8	90.4	91.9	71.1	20.02 M	10.24
②	√	-	-	-	91.4	92.3	93.2	73.3	16.95 M	8.16
③	√	√	-	-	91.7	92.5	93.8	74.2	17.03 M	8.19
④	√	√	√	-	95.3	92.4	95.4	77.3	17.20 M	8.30
⑤	√	√	√	√	95.5	93.6	96.1	78.4	17.20 M	8.30

**Table 9:** Model comparison results.

Methods	Precision (%)	Recall (%)	mAP@50 (%)	mAP@50:95 (%)	Params	GFLOPs
Faster R-CNN	75.7	73.9	76.5	51.2	62.66 M	24.12
YOLOv5	85.9	83.6	86.1	59.6	55.45 M	20.42
YOLOv8	84.5	82.6	84.7	58.0	52.12 M	18.98
YOLOv10	89.9	90.8	91.1	68.5	22.12 M	10.03
YOLOv11	90.1	89.5	91.3	67.1	23.84 M	12.25
YOLOv12	89.8	89.7	91.3	66.4	22.72 M	13.67
MobileNetv4-YOLOv11	82.9	84.4	85.9	60.3	15.62 M	6.87
YOLO-nano	73.0	70.9	73.2	48.9	8.59 M	4.51
RT-DETR	90.8	90.4	91.9	71.1	20.02 M	10.24
SDD-RT-DETR	95.5	93.6	96.1	78.4	17.20 M	8.30

## Data Availability

The source code of SDD-RT-DETR is publicly available at https://github.com/zach662/SDD-RT-DETR-scallop-doneness-detection (accessed on 1 June 2026).
